# Rapid Analyses of Polyetheretherketone Wear Characteristics by Accelerated Wear Testing with Microfabricated Surfaces for Artificial Joint Systems

**DOI:** 10.1155/2017/5979564

**Published:** 2017-11-02

**Authors:** Chen-Ying Su, Chien-Wei Kuo, Hsu-Wei Fang

**Affiliations:** ^1^Department of Chemical Engineering and Biotechnology, National Taipei University of Technology, No. 1, Sec. 3, Zhongxiao E. Rd., Taipei 10608, Taiwan; ^2^Institute of Biomedical Engineering and Nanomedicine, National Health Research Institutes, No. 35, Keyan Road, Zhunan Town, Miaoli County 35053, Taiwan

## Abstract

Wear particle-induced biological responses are the major factors resulting in the loosening and then failure of total joint arthroplasties. It is feasible to improve the lubrication and reduce the wear of artificial joint system. Polyetheretherketone (PEEK) is considered as a potential bearing material due to its mechanical characteristics of resistance to fatigue strain. The PEEK wear particles have been indicated to be involved in biological responses in vitro, and further studies regarding the wear phenomena and wear particle generation are needed. In this study, we have established an accelerated wear testing system with microfabricated surfaces. Various contact pressures and lubricants have been utilized in the accelerated wear tests. Our results showed that increasing contact pressure resulted in an increase of wear particle sizes and wear rate, and the size of PEEK wear particles can be controlled by the feature size of microfabricated surfaces. These results provided the information rapidly about factors that affect the morphology and amount of PEEK wear particles and can be applied in the future for application of PEEK on the biological articulation system.

## 1. Introduction

Many reasons can cause the damage of joints such as aging, stress, trauma, or body weight. When the joints are damaged severely resulting in pain and even difficulties in movements, total joint arthroplasties (TJAs) are recognized as the ultimate effective treatment currently. The performance of TJAs depends on their tribological properties, and the wear particles that are generated from the tribological process play a key role in the lifetime of artificial joint system [1]. It has been shown that wear particles induce biological responses and even cause osteolysis and bone resorption [2]. One of polymers, polyetheretherketone (PEEK), has been introduced as bearing materials for TJAs because of its resistance to fatigue strain [3, 4]. Therefore, it is critical to prove whether PEEK wear particles could induce biological responses in vivo.

The current method of generating wear particles is employing joint stimulators, which are cost- and time-consuming [5]. Moreover, many factors can affect the wear rate including contact pressures, lubricants, sliding conditions, or surface geometries and it is difficult to investigate many factors by joint simulators [6]. The wear rate is correlated with the sizes and morphology of wear particles, and it has been shown that the size of PEEK wear particles is responsible for inducing biological responses [7]. Therefore, a rapid method of generating various wear particles for investigating PEEK particle-induced biological responses is required.

**Figure 1:**
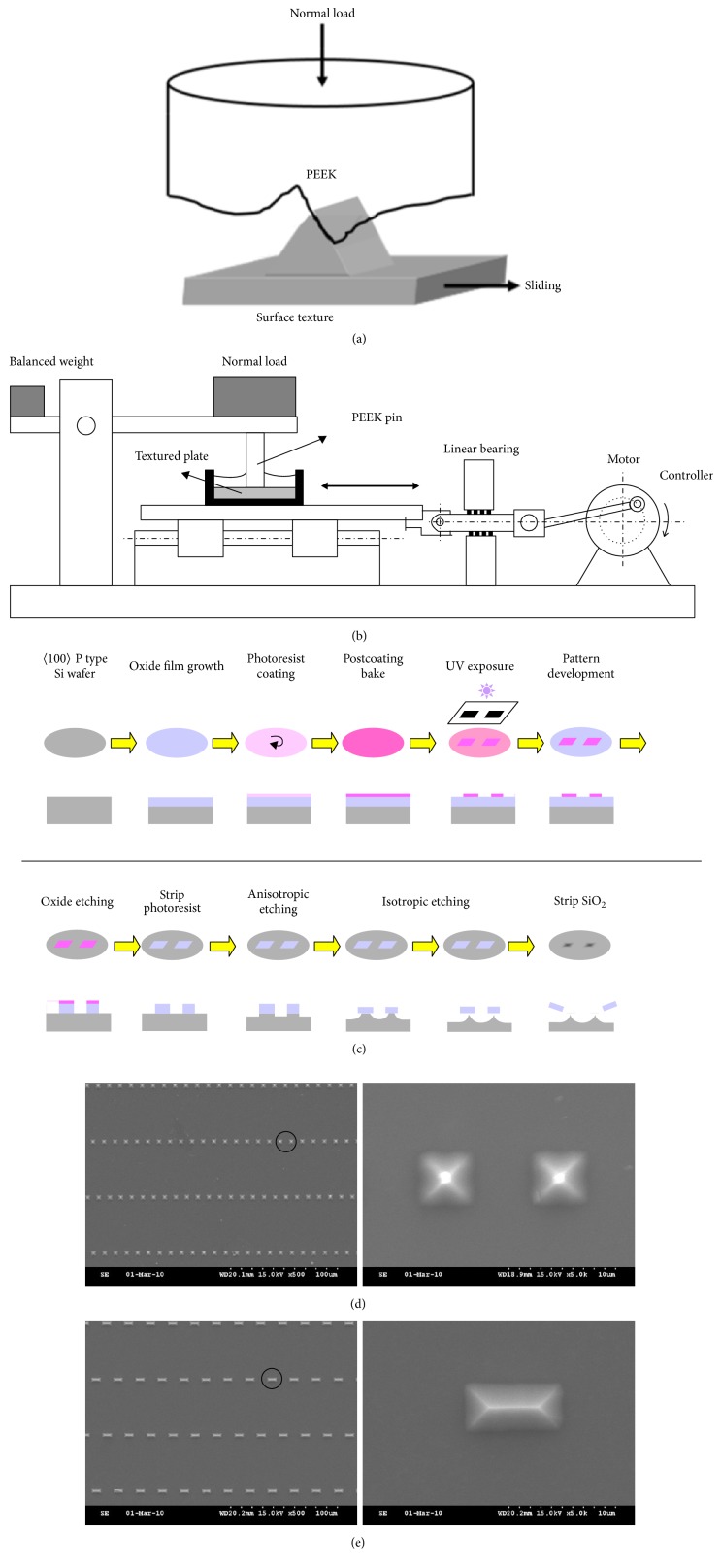
*Experimental setup of wear testing.* (a) Schematic of the particle generation in which microfabricated surface texture is rubbed against PEEK pin. (b) Schematic of the setup for linear reciprocating wear testing. (c) Schematic of the microfabrication process of surface textures on the silicon wafer. (d) SEM images of the surface S; the wedges are 5 *μ*m in width and 5 *μ*m in length. The textures in the black circle are magnified on the right. (e) SEM images of the surface L; the wedges are 5 *μ*m in width and 10 *μ*m in length. The textures in the black circle are magnified on the right.

In this study, we developed an accelerated wear testing protocol to investigate the effects of different contact pressures and different lubricants on the wear rate and morphology of PEEK particles. By rubbing a PEEK pin on different dimensions of the microfabricated surfaces (Figure 1(a)), the relationship between particle morphology and surface geometries was evaluated. Our results demonstrated a rapid method for analyzing factors that may affect the characteristics of PEEK wear particles.

## 2. Material and Methods

### 2.1. Materials

PEEK cylinder pins were obtained from A-SPINE Asia Co. Ltd. (batch number was SSR 0151). The pins were 6.35 mm in diameter and 25.4 mm in length with diamond turning on both end surfaces without polishing. Fetal bovine serum (FBS, Hyclone) was diluted in phosphate buffered saline (PBS) to 25%. 50% and 10% of glycerine (J.T.Baker) was prepared in distilled water. All PEEK pins were presoaked in PBS for at least 30 days so as to become completely saturated.

### 2.2. Wear Process

By rubbing PEEK pin with the cutting edges of the silicon surface textures, the PEEK wear particles were generated. ASTM F732 was used as a guideline. The setup of the system was described [8], and the relative motions between a PEEK pin and a cutting device were illustrated in Figure 1(b). The PEEK pin was weighed three times and mounted on the tester. Linear reciprocating wear tests were run under a nominal contact pressure of 0.6, 1.5, or 3.0 MPa, a stroke length of 19 mm, and an average sliding speed of 57.2 mm/s for 6 hours. After wear process, the PEEK pin was weighed and the wear loss was obtained after adjusting the weight change from before test.

### 2.3. Microfabricated Surface Textures

The silicon wafer surface with controlled asperities was prepared by photolithography patterning and etching of the bulk substrate as shown in Figure 1(c) and previously described [9]. Two-inch diameter polished type P silicon wafers with (100) orientation purchased from Summit-Tech were used as the substrate material. Wet oxidation of the silicon wafers was carried out in a glass-tube oven at 1100°C for 135 minutes to form a silicon dioxide film with a thickness of 1 *μ*m. A pattern of rectangles with different size and aspect ratios was made on a chrome direct-writing photomask. The silicon dioxide (SiO_2_) surface was spin coated with a Shipley 1813 positive photoresist. The dark-featured photomask with rectangular patterns (5 *μ*m × 5 *μ*m, hereafter called surface S; 5 *μ*m × 10 *μ*m, hereafter called surface L) were then placed on the photoresist surface and exposed to an ultraviolet source in a mask aligner to decompose the surrounding polymer surface, leaving a positive rectangular pattern. The decomposed photoresist polymer was then removed in a Shipley 351 developer. The resulting positive photoresist surface was then etched first with a buffered oxide etch (BOE) solution (diluted buffer hydrofluoric acid (HF) solution) to etch away the SiO_2_ in a wet chemical bath. The photoresist was then removed by washing with acetone-alcohol. Subsequently, the silicon material was subjected to isotropic silicon etching (HNA etchant; liquid volume ratio HF : HNO_3_ : CH_3_COOH = 8 : 75 : 17) in a wet chemical bath at room temperature. The SiO_2_ layer was removed after an isotropic undercutting etching process. The resulting surface features are an array of rectangular ridges with sharp edges. Finally, a layer of 5 nm Cr coating was evaporated onto the surface to increase the strength and wear resistance of the surface texture. The height of the surface textures was measured by a Mahr profilometer (Gottingen, Germany) and the feature length and width were measured from scanning electron microscopy (SEM) observations.

### 2.4. Isolation of the Wear Particles

PEEK particles were collected in a sterilized beaker. The collected solution was added with 6 N NaOH and shaken at 65°C for 24 hours. After centrifuging, collecting the bottom solid layer, adding new NaOH, and shaking at 65°C for 24 hours, the solution was again centrifuged at 5000 rpm at 4°C for 1 hour. The bottom layer was rinsed with 50 mL purified water, sonicated for 10 minutes, and then centrifuged at 5000 rpm at 4°C for 30 minutes. Particles were rinsed with purified water twice and then once with 95% ethanol. All the liquid was discarded, and 95% ethanol was added. After sonicating for 10 minutes, wear particles were collected on a 0.1 *μ*m pore size membrane through a vacuum filtration process.

### 2.5. Analysis of the Particles

The particles collected on the filter paper were examined by using a scanning electron microscope. Micrographs of the particles were then analyzed by using image analyzer software (Scion Image, a personal computer version of NIH Image) to measure their dimensions. Measurements were made for at least 300 particles in each condition.

## 3. Results and Discussion

### 3.1. Decreasing Contact Pressure Results in Reducing PEEK Wear Particle Length and Wear Rate

**Figure 2:**
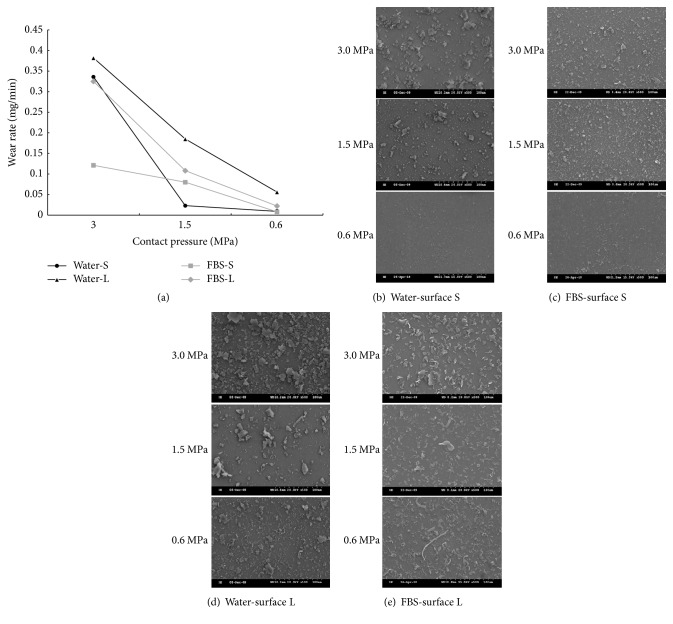
*Morphology of wear particles under the lubrication of water and FBS.* (a) Plots of the wear rate in water or FBS versus different contact pressures. (b) and (c) SEM images of the PEEK wear particles by rubbing PEEK pin against surface S in water (b) or in FBS (c) under the contact pressure of 3.0, 1.5, and 0.6 MPa. (d) and (e) SEM images of the PEEK wear particles by rubbing PEEK pin against surface L in water (d) or in FBS (e) under the contact pressure of 3.0, 1.5, and 0.6 MPa.

The PEEK wear particles produced by rubbing PEEK pin in water and tested with three different contact pressures were analyzed (Table 1). We found that decreasing contact pressure resulted in a reduction of particle length, particle width, and wear rate (Figure 2(a) and Table 1). We also observed that, under the same contact pressure, rubbing PEEK pins against the surface L led to a larger particle length, width, and wear rate compared to the surface S (Figure 2(a) and Table 1). In addition, we observed that rubbing PEEK pins against surface L caused sheet-like particles while rubbing against smaller surface resulted in granular-like particles (Figures 2(b) and 2(d)).

The articulation of PEEK pins were also carried out in FBS, which is widely used in joint stimulations. Similar outcomes were observed: decreasing contact pressure caused a reduction in particle length, particle width, and the wear rate (Table 1). We observed both long fibrous and thin platelet wear particles in FBS regardless of contact pressures and surface geometries (Figures 2(c) and 2(e)) that was distinct from rubbing in water, this phenomenon only observed in more lubricating environment [6].

When we compared the wear rate with water and FBS against the same surface geometry, we found that the wear rate was smaller when articulation of PEEK pin was in FBS than in water regardless of the contact pressures (Figure 2(a)). Indeed, FBS contains many components such as albumin, globulin, and glucose that are similar to human synovial fluid. Interestingly, the shapes of wear particles in FBS were distinct compared with them being in water. It is possible that FBS forms a thin layer on the surfaces to increase the modulus of elasticity of PEEK; thus PEEK was harder to be broken resulting in fibrous shape of particles especially under a smaller contact pressure.

### 3.2. Effects of Glycerine on Wear Particle Length and Amounts

**Figure 3:**
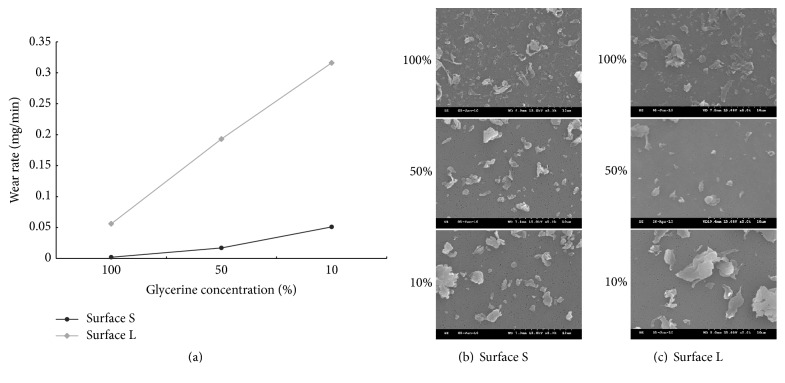
*Morphology of wear particles under the lubrication of glycerine.* (a) Plots of the wear rate versus different concentrations of glycerine. (b) and (c) SEM images of the PEEK wear particles by rubbing PEEK pin against surface S (b) or surface L (c) under the contact pressure of 1.5 MPa in 100%, 50%, and 10% of glycerine.

We next investigated whether changing the concentration of a specific lubricant has effects on particle size and wear rate. Our results demonstrated that decreasing the concentration of glycerine resulted in an increase in particle length, width, and wear rate (Figure 3(a) and Table 1). We also noticed that the morphology of wear particles were both sheet-like and granular-like in 100% glycerine (Figures 3(b) and 3(c)). In contrast, the wear particle shape in 10% glycerine was uniformly sheet-like regardless of the surface geometries suggesting that 100% glycerine provided a more lubricating environment.

Glycerine is viscous liquid, and our results showed that high viscosity of glycerine caused the smallest particles and the wear rate (Table 1). It has been shown that the higher viscosity of hyaluronic acid results in fewer wear amounts and smaller particle size, and it may explain the outcomes in our observation [10]. Both rod-like and granular shape of carbon fiber reinforced-PEEK (CFR-PEEK) wear particles were identified in macrophages in human tissue retrieval studies [11, 12], although the effects of particle shapes on the artificial joint system are still unclear. Therefore, the method we developed here could be rapidly generating various shapes of PEEK wear particles for the further investigation.

## 4. Conclusion

An accelerated wear testing procedure was established successfully to investigate the effects of contact pressures, surface geometries, and lubricants on the characteristics of PEEK wear particles. This accelerated wear testing with microfabricated surfaces can facilitate the generation of specific sizes or shapes of PEEK wear particles in order to study PEEK wear particle-induced biological responses in vivo.

## Figures and Tables

**Table 1:** Morphology and wear of PEEK particles in water, FBS, and glycerine.

Lubricant	Surface	Contact pressure (MPa)	Particle length (*μ*m)	Particle width (*μ*m)	Aspect ratio	Wear rate (mg/min)
Water	S	3.0	1.85 ± 0.80	1.22 ± 0.54	1.56 ± 0.36	0.336
		1.5	1.40 ± 0.64	0.92 ± 0.41	1.56 ± 0.39	0.023
		0.6	1.24 ± 0.54	0.85 ± 0.36	1.51 ± 0.38	0.009
	L	3.0	2.80 ± 1.53	1.65 ± 0.81	1.74 ± 0.54	0.382
		1.5	3.13 ± 1.75	1.72 ± 1.03	1.95 ± 0.73	0.185
		0.6	2.29 ± 1.31	1.06 ± 0.52	2.27 ± 1.07	0.056

FBS	S	3.0	1.29 ± 0.60	0.77 ± 0.33	1.71 ± 0.46	0.121
		1.5	0.99 ± 0.41	0.57 ± 0.23	1.77 ± 0.48	0.08
		0.6	0.81 ± 0.34	0.48 ± 0.22	1.85 ± 0.60	0.008
	L	3.0	9.06 ± 3.05	5.16 ± 2.05	1.89 ± 0.74	0.325
		1.5	6.28 ± 2.68	3.75 ± 1.57	1.75 ± 0.57	0.108
		0.6	3.77 ± 1.84	1.98 ± 1.32	1.91 ± 0.91	0.022

100% Glycerine	S	1.5	0.50 ± 0.31	0.28 ± 0.18	1.83 ± 0.63	0.002
50% Glycerine			0.82 ± 0.41	0.53 ± 0.25	1.60 ± 0.44	0.017
10% Glycerine			1.08 ± 0.59	0.73 ± 0.40	1.53 ± 0.36	0.051
100% Glycerine	L		0.65 ± 0.54	0.39 ± 0.28	1.73 ± 0.57	0.056
50% Glycerine			1.58 ± 1.22	0.91 ± 0.60	1.76 ± 0.56	0.193
10% Glycerine			2.16 ± 1.26	1.13 ± 0.62	2.02 ± 1.02	0.316
